# Green and Hawksbill Sea turtles of Eastern Atlantic: New insights into a globally important rookery in the Gulf of Guinea

**DOI:** 10.1002/ece3.11133

**Published:** 2024-03-18

**Authors:** Betânia Ferreira‐Airaud, Sara Vieira, Maria Branco, Antunes Pina, Venceslau Soares, Manjula Tiwari, Matthew Witt, Rita Castilho, Alexandra Teodósio, Lucy A. Hawkes

**Affiliations:** ^1^ Centro de Ciências do Mar (CCMAR) Universidade do Algarve Faro Portugal; ^2^ Hatherly Laboratories University of Exeter Exeter UK; ^3^ Programa Tatô São Tomé São Tomé and Príncipe; ^4^ Ocean Ecology Network Research Affiliate of NOAA Southwest Fisheries Science Center La Jolla California USA

**Keywords:** conservation, gulf of Guinea, marine protected areas, nesting behaviour, satellite telemetry, sea turtles

## Abstract

Sea turtles are critical components of marine ecosystems, and their conservation is important for Ocean Governance and Global Planet Health. However, there is limited knowledge of their ecology in the Gulf of Guinea. To fill this knowledge gap, this study presents the first integrative assessment of green and hawksbill turtles in the region, combining nesting surveys over 9 years and telemetry data, to offer insights into these population dynamics, and behaviours, including nesting preferences, morphological and reproductive parameters, diving patterns and inter‐nesting core‐use areas. Both green and hawksbill turtles are likely making a recovery on São Tomé, potentially driven by sustained conservation efforts. There are preliminary indications of recovery, but we interpret this cautiously. Coupled with satellite tracking, this study estimated that 482 to 736 green turtles and 135 to 217 hawksbills nest on the beaches of São Tomé. Their movements overlap significantly with a proposed Marine Protected Area (MPA), which suggests they may be well placed for conservation if managed appropriately. However, the presence of artisanal fisheries and emerging threats, such as sand mining and unregulated tourism, highlight the urgent need for robust management strategies that align global conservation objectives with local socioeconomic realities. This study significantly enhances our understanding of the ecology and conservation needs of the green and hawksbill turtles in the Gulf of Guinea. The insights gleaned here can contribute to the development of tailored conservation strategies that benefit these populations and the ecosystem services upon which they depend.

## Graphical abstract

This study presents a comprehensive assessment of green and hawksbill sea turtles in the Gulf of Guinea, combining 9 years of nesting area surveys with telemetry data. Our findings reveal promising signs of recovery for green and hawksbill turtles, driven by conservation efforts. Our research underscores the importance of effective conservation strategies, especially in the face of emerging threats, to protect these vital marine species and their ecosystems in the Eastern Atlantic.

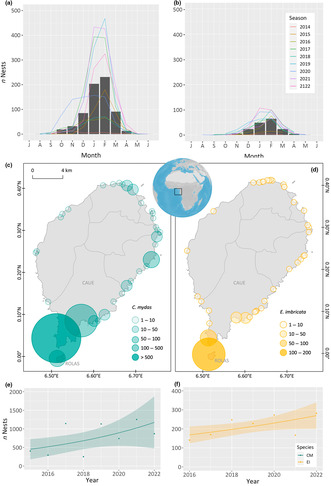

## 1 INTRODUCTION

Marine biodiversity faces unprecedented pressures from anthropogenic activities, particularly regulated and unregulated commercial and artisanal fishing (FAO et al., 2023), as well as marine traffic, pollution and climate change (Duarte et al., 2020; Halpern et al., 2019; Lotze, 2021). Anthropogenic threats often overlap with ecologically important areas used by marine migratory vertebrate species, such as seabirds, mammals, sharks, and sea turtles (Lascelles et al., 2014; McCauley et al., 2015; Polidoro et al., 2017; Sequeira et al., 2019). Many of these taxa face significant reductions in their population numbers and are at risk of extinction (Butchart et al., 2010; Dulvy et al., 2021; Mortimer & Donnelly, 2008; Pimiento et al., 2020; Restrepo et al., 2023; Wallace et al., 2011), requiring ocean governance with conservation measures (Dulvy et al., 2021; Pimiento et al., 2020) that range from intergovernmental agreements to national and local conservation programs, including protected areas (Duda, 2016; Huang et al., 2022; Lotze, 2021; Maestro et al., 2019). Even when some sea turtle populations are showing signs of recovery (Mazaris et al., 2017), significant conservation concerns persist, underlining the need for a nuanced understanding of specific regional and species characteristics. In this context, green (*Chelonia mydas*) and hawksbill (*Eretmochelys imbricata*) sea turtles nesting in São Tomé and Príncipe, a region with unique ecological attributes and conservation challenges (Hamann et al., 2010; Hancock et al., 2019; Monzón‐Argüello et al., 2011; Wallace et al., 2011), present an interesting case study.

São Tomé and Príncipe is a small island country in the Gulf of Guinea in the Eastern Tropical Atlantic. Composed of two main islands and several islets, this archipelago is considered a marine biodiversity hotspot (Roberts et al., 2002) and a globally important region, hosting high concentrations of rare and threatened marine species (Polidoro et al., 2017), such as sharks and rays (Moreira da Costa et al., 2022), marine mammals (Carvalho et al., 2022) and important breeding and foraging grounds for five of the seven species of sea turtles, all of which are listed on the IUCN Red List of Threatened Species (the green, the hawksbill, the olive ridley (*Lepidochelys olivacea*), the leatherback (*Dermochelys coriacea*), and the loggerhead (*Caretta caretta*)) (Castroviejo et al., 1994; Dontaine & Neves, 1999; Ferreira‐Airaud et al., 2022; Formia et al., 2003; Fretey, 2001; Graff, 1996). Historical records reveal that sea turtles were once abundant in São Tomé and Príncipe (Matos, 1916) and might have been heavily exploited for their meat and shell since the 16th century, when these islands were first inhabited (Greeff, 1884; Parsons, 1962, 1972). Until very recently, turtles were captured for their meat and eggs, and hawksbill turtle carapaces were collected for the manufacture of handicrafts and jewellery – possibly the greatest driver of their decline in the country (Castroviejo et al., 1994; Ferreira‐Airaud et al., 2022; Fretey, 1998). São Tomé and Príncipe host the last remaining significant hawksbill turtle nesting aggregation in the Eastern Atlantic and ranks among the top 11 sea turtle conservation priorities worldwide (Hamann et al., 2010; Wallace et al., 2011). Hawksbill turtles have been documented to nest sporadically along the West and Central African continental coasts in small numbers in Guinea Bissau (an estimated 20 nests annually, Barbosa et al., 2018); Gabon (an average of 7 nests annually, Girard et al., 2016); and Bioko (an estimated 10 nests annually, Honarvar et al., 2016). However, in São Tomé and Príncipe, between 70 and 350 nests have been estimated annually between both islands (Ferreira‐Airaud et al., 2022). Hawksbill turtles nesting here have a unique genetic haplotype and low genetic variability, which emphasises this population's high degree of isolation and vulnerability (Monzón‐Argüello et al., 2010, 2011). In contrast, green turtles exhibit a more extensive nesting distribution throughout the Eastern Atlantic, with the largest known rookery occurring in Guinea‐Bissau (with over 25,000 nests laid annually, Patrício et al., 2019), followed by São Tomé and Príncipe (between 330 and 3200 nests laid annually on both islands, Ferreira‐Airaud et al., 2022) and Bioko (between 170 and 1830 estimated nests laid annually, Honarvar et al., 2016). More nesting occurs sporadically along the rest of the West African coast, but much is unconfirmed or limited in numbers (Oliwina et al., 2020). The green turtle nesting population of São Tomé and Príncipe is also considered a genetically distinct rookery (Formia et al., 2006; Hancock et al., 2019), exhibiting relatively high levels of genetic diversity and distinctiveness and representing an important genetic pool in the region (Hancock et al., 2019).

While São Tomé and Príncipe islands are recognised as key nesting sites for sea turtles in the Eastern Atlantic, a comprehensive understanding of this region's green and hawksbill turtle populations remains elusive. The existing gaps in knowledge about their biology, ecology and behaviour, temporal and spatial distributions, and habitat use, especially away from the nesting beaches, hinder the development of effective conservation strategies. In this study, we evaluate trends in the green and hawksbill turtle nesting populations to determine if there are signs of recovery following recent conservation measures undertaken in the country. Additionally, we characterise their nesting site fidelity and use of coastal areas during their inter‐nesting interval, which is critical for determining the minimal protected area necessary for effective species conservation. Our study combines multiple methods, including nesting surveys, satellite tracking data and diving data, to provide a comprehensive overview of green and hawksbill nesting turtles' population behaviour and habitat use in São Tomé waters for the first time. By contrasting turtle habitat use with small‐scale fishing activity and proposed marine protected areas, we aim to inform and enhance national conservation efforts.

## 2 MATERIALS AND METHODS

### 2.1 Study area

São Tomé and Príncipe, a small island nation off the Western Central Coast of Africa (0.263584° N, 6.602234° E), comprises two equatorial oceanic islands, São Tomé (857 km^2^) and Príncipe (139 km^2^) and several small islets (Ceríaco et al., 2022). It is part of the Cameroon Volcanic Line, an extinct volcanic mountain chain in the Gulf of Guinea (Alegre, 2009; Bollen, 2017; Ceríaco et al., 2022). São Tomé is home to about 215,000 inhabitants, 96% of the nation's population (INE‐STP, 2020), and is surrounded by shallow rocky reefs with hard and soft corals, vast beds of rhodoliths, macroalgae and seagrass meadows. In the northeastern region of São Tomé, the coastline ranges from extensive shallow shelves while the southern coast is surrounded by deeper water and is more exposed to waves. There are estuarine and mangrove habitats bordering the island (Airaud et al., 2020; Ferreira‐Airaud et al., 2021), along with 112 beaches ranging from golden yellow to dark grey sand and shingle beaches. While our research was conducted within the São Tomé and Príncipe archipelago, it is important to note that this study specifically focused on nesting sites located on the island of São Tomé. Although Príncipe also hosts important sea turtle nesting sites, our data collection did not extend to this island. However, for a holistic understanding of the sea turtle nesting dynamics in the region, it is recognised that Príncipe likely accommodates approximately 30% of the hawksbill and 65% of the green turtle nesting populations in the archipelago (Ferreira‐Airaud et al., 2022).

### 2.2 Nesting beach classification and monitoring effort

Sea turtle conservation efforts were initiated in the late 90s in São Tomé (Ferreira‐Airaud et al., 2022), but reliable data are only available from 2014 onwards, when consistent monitoring efforts and data collection were initiated. Both green and hawksbill turtles typically nest between September and April, but the nesting season was defined as the period running from 1 July of 1 year to 30 June of the following year to accommodate the majority of the observed nesting events. São Tomé has 112 beaches classified according to nest density: absent or rare (32 beaches); and very low, low, medium and high (80 beaches cumulatively). This classification was based on the mean annual number of nests observed per beach, providing a systematic approach to prioritise and allocate resources for surveys and conservation efforts. Absent or rare classification indicates no nests or sporadic nests; very low indicates less than ten nests per beach; low indicates 10 to 50 nests per beach; medium, 50 to 100; and high, more than 100 nests per beach. Most beaches are relatively small (between 0.02 and 2.24 km long), but they are often far apart, requiring significant logistical effort and resources to survey. Weekly censuses were performed along 29 very low or low nesting density beaches (total 8 km) between 1 November and 28 February of every year (i.e., the months with the highest nest density). Daily censuses were performed along the remaining 51 beaches (totalling 15 km) between 1 September and 30 April of every year (the nesting season duration) to record nesting activity from the preceding night, mark nests and monitor them until hatching occurred. Together with these daily censuses, night patrols were also undertaken every night on the same 51 beaches to collect data on nesting turtles and their nests, as well as to prevent and deter illegal harvesting.

### 2.3 Nesting data collection and analysis

We recorded nest parameters, whenever possible, including clutch size, nest depth, emergence success and incubation period. Females were tagged during nesting with Inconel tags (Style 681, National Band and Tag Company) in both front flippers, and their curved carapace length (CCL) was measured. To analyse CCL changes between nesting seasons, we used a Kruskal‐Wallis test. For the CCL analyses, we considered only the first recorded data from each female per season to avoid potential bias introduced by repeat records of high‐frequency nesters, ensuring that each turtle contributed only once to the data set within a nesting season. For other reproductive parameters such as clutch size, nest depth, emergence success and incubation period, we conducted a descriptive statistical analysis using all available data from each female throughout the season. This involved calculating the mean, minimum, maximum and standard deviation for each parameter to provide a comprehensive understanding of their distribution and variability across the nesting season. We calculated emergence success as the proportion of eggs that produced live hatchlings that reached the surface of the sand and included the failed nests (with 0% emergence success). The incubation period was determined as the number of days between the day of oviposition and the emergence of the first hatchling.

### 2.4 Population size and trend estimation

Inter‐nesting intervals were calculated as the number of days between observed nesting events. In some cases, females were observed re‐nesting after intervals corresponding to two or three inter‐nesting periods, as identified by the multimodal pattern in our data, suggesting that they had laid one or more unobserved clutches during these intervals. We corrected the Observed Clutch Frequency (OCF) to account for these potentially missed nests, referred to as Estimated Clutch Frequency (ECF; Frazer & Richardson, 1985; Johnson & Ehrhart, 1996). Remigration intervals were calculated as the duration in months between nesting events in successive years. Inter‐nesting intervals, ECF and remigration intervals were then used to estimate the number of individual females contributing to the nesting populations of each species. This approach likely better accounts for missed nests than direct counting of nesting turtles. Due to the relatively small hawksbill population in São Tomé, the dataset describing their nesting is limited, and this should be taken into consideration when interpreting the remigration interval data for this species.

To assess the trends in nesting numbers for green turtles and hawksbill sea turtles, we analysed the data recorded from the nesting seasons from 2015 to 2022. This analysis period was selected to ensure consistency in survey methodology and coverage, as the data from 2014 for green turtles and 2014 and 2015 for hawksbill turtles were excluded due to lower survey effort and area coverage in these initial years. We employed a Negative Binomial regression analysis to evaluate the trends in the number of nests using the glm.nb function from the MASS package in R (version 2023.03.0 + 386). This method allowed us to more accurately model the count nature of our nesting data, providing a robust analysis of trends over the study period. Nest counts over the nine‐year study period were based on data from daily and weekly monitored beaches, in line with their nesting density classification. Medium‐ and high‐density beaches were monitored daily, ensuring accurate recording of each nesting event or false crawl. Weekly monitoring on very low and low‐density beaches could miss some nests due to high tides, rain or other natural occurrences, but old nests were often identified and monitored until emergence to confirm species. Despite less frequent monitoring, these counts provide a reliable nesting event estimate, contributing to accurate season nest number estimation.

### 2.5 Turtle capture and satellite tag deployment

Satellite tags were deployed on eight green turtles nesting at Jale beach and 17 hawksbill turtles nesting at Joana and Marinho beaches at Rolas Islet. Praia Jalé is a high‐energy beach with a narrow shelf where most green turtles nest, along with leatherback and hawksbill turtles. Rolas Islet is a small rocky islet in southern São Tomé and is the main nesting site for hawksbill turtles and some green turtles (Ferreira‐Airaud et al., 2022). To collect comprehensive data on the nesting behaviour and movement and diving patterns of the turtles during the inter‐nesting period, we deployed two different models of satellite tags: the SPLASH10‐F‐385A and the SPOT‐375B, manufactured by Wildlife Computers. The SPLASH10‐F‐385A, deployed on 15 turtles (5 green and 10 hawksbills), transmits Argos and Fastloc GPS data, providing high‐resolution spatial data and recording vertical movements. The SPOT‐375B, deployed on 10 turtles (3 green and 7 hawksbills), transmits only Argos data, offering valuable information on wider‐scale movements. Satellite tags were deployed on nesting females during nightly surveys in the period of highest nest density for both species between 11 December 2019 and 16 January 2020 and between 26 January 2021 and 3 February 2021. Nesting females were intercepted after they finished egg‐laying and instrumented with transmitters using established protocols (Wildlife Computers, 2019a, 2019b).

### 2.6 Tracking data filtering

SPLASH tags were programmed to continuously collect location data, and nesting events were detected from the tags' wet/dry sensor. The last haul‐out position represented the nesting event when subsequent haul‐outs were detected on consecutive days or even the same night. As soon as the turtles made continuous directional and persistent movements away from the coast, it was considered the completion of nesting and the start of the post‐nesting migration. For SPOT tags, only high quality and accurate locations were retained for analyses, filtering raw location data to retain only the location classes 3 and 2, which have radial spatial errors of <500 m (CLS, 2016). Fastloc‐GPS data was subjected to a different filter, retaining only locations derived from six or more satellites (Witt et al., 2010). This rigorous data filtering allowed us to construct reliable and accurate evidence of turtle movements.

### 2.7 Population size estimation with satellite tracking

In estimating population size using satellite tracking, the haul‐out data provided by the satellite tags was instrumental in determining nesting events with certainty, allowing for accurate calculations of inter‐nesting intervals and clutch frequencies. These were then cross‐verified with field observations. This approach was supplemented by our continuous monitoring of nesting beaches from the season's onset and tagging efforts, ensuring broad coverage of the nesting females nesting in the study area. Although we installed satellite tags predominantly around the peak of the nesting season, our comprehensive beach monitoring prior to this period assures us that the impact of potentially unobserved early clutches on our estimates is minimal. Nonetheless, we recognise this as a limitation and have taken it into account in our analysis. The combined use of satellite tracking data and field observations, including early season monitoring, enabled us to estimate the total number of females for each species while acknowledging the inherent uncertainties in both methods.

### 2.8 Spatial analysis and habitat use assessment

Due to the high spatial accuracy of Fastloc‐GPS tags compared to Argos tags, only Fastloc‐GPS tags were used to estimate the spatial distribution of both green and hawksbill turtles during the inter‐nesting period. The 50, 75 and 90% home ranges of tracked female turtles were estimated using autocorrelated kernel density estimation (AKDE) in the continuous‐time movement modelling framework (R package ctmm; Calabrese et al., 2016). The minimum convex polygons (MCP) were calculated in QGIS‐LTR (version 3.16.16‐Hannover). Special attention was given to exclude terrestrial overlaps from the analysis, ensuring that the area calculations represent exclusively the marine habitats utilised by the turtles. Bathymetry contours polygons were created using data from the hydrographic chart of São Tomé (Instituto Hidrográfico de Portugal, 1970). Additionally, to provide a more comprehensive understanding, marine coastal habitat information previously developed by the author was integrated into the analysis (see Airaud et al., 2020). Through participatory agreements, we also integrated data collected between 2016 and 2018 regarding the location of fishing vessels during fishing activity into our spatial analyses. We fitted GPS trackers on 25 boats of fishers, programmed to record GPS locations every 5 min throughout an entire nesting season. Based on the findings from Ferreira‐Airaud et al. (2022), we incorporated fishing gears into our analysis that were identified as having the highest potential to impact sea turtles – these included purse‐seine, longline, troll line, and surface and bottom gillnets. The proposed boundaries of a Marine Protected Area (MPA) south of São Tomé (Jalé‐Rolas‐Malanza) were also integrated into the spatial analyses to determine the percentage of sea turtle locations inside and outside the proposed MPA.

Turtle diving data gathered by SPLASH tags were analysed using R (version 4.1.3) and RStudio (version 2022.02.3 Build 492). For each species, we calculated dive depths (in meters) and durations (in minutes), summarising this information as a time‐at‐depth distribution across ten depth bins. The upper limits of the depth bins were set at 0, 5, 10, 15, 20, 25, 30, 35, 40 and 45 metres, while the duration bins were capped at 0, 5, 10, 20, 30, 40, 60, 80, 100 and 120 min. Additionally, we retrieved complete archival data from the satellite transmitters of two individuals. This allowed for a more detailed analysis of diving behaviour at the level of individual dives for these specific turtles, enriching our understanding of their diving patterns in contrast to the collective analysis conducted for the rest of the tagged turtles.

## 3 RESULTS

### 3.1 Spatial and temporal distribution of nests and population trends

**FIGURE 1:**
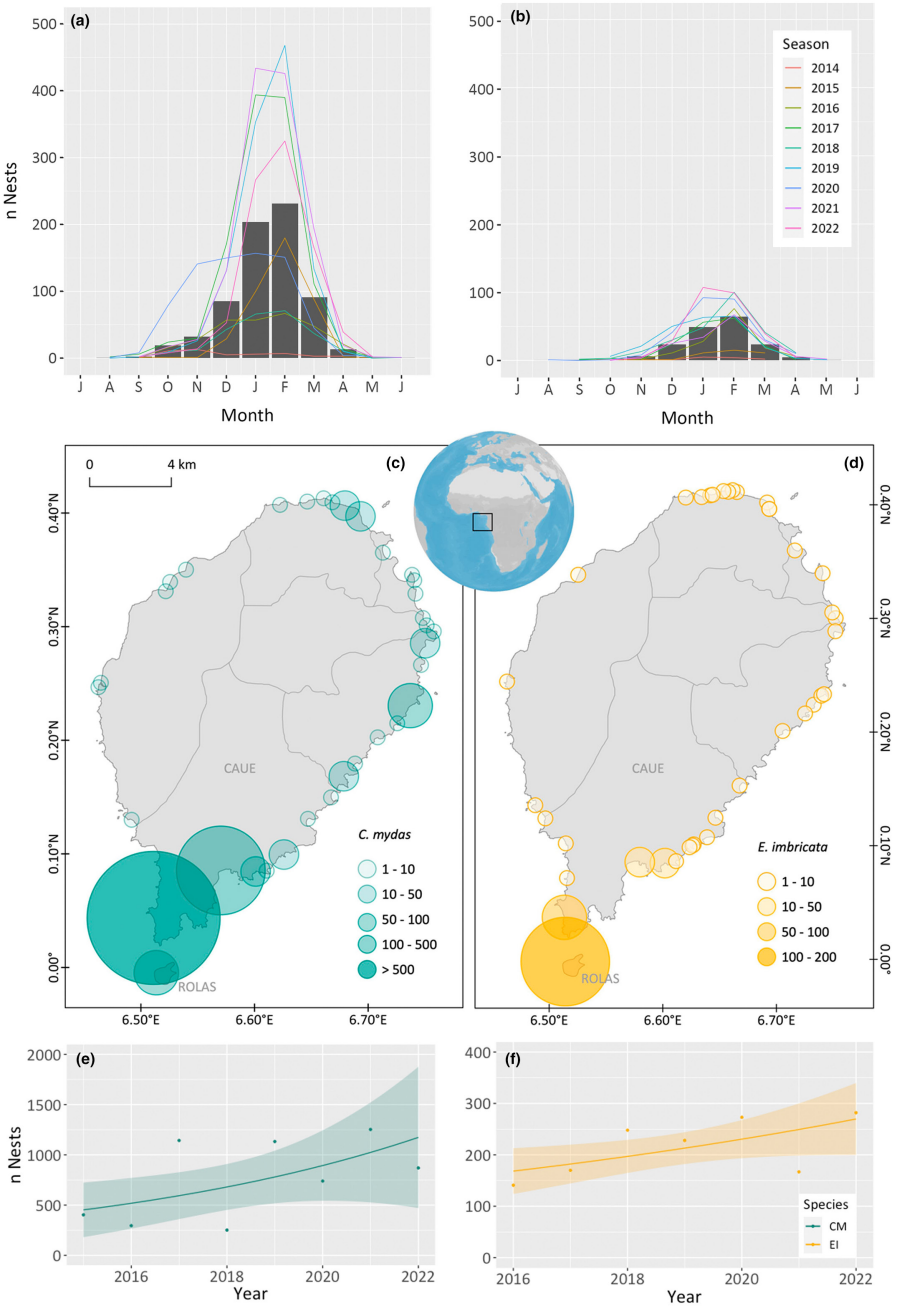
Number of nests per season (2014 to 2022) throughout the nesting season from July (J) to June (J) of the following year for (a) green turtles and (b) hawksbill turtles nesting in São Tomé. Distribution of the mean number of nests from 2014 to 2022 in São Tomé, for (c) green (*Chelonia mydas*) and (d) hawksbill (*Eretmochelys imbricata*): 1–10 – very low nest density; 10–50 – low nest density; 50–100 – medium nest density, and more than 100 high nest density. Trend in the number of nests laid by both species (e) green (CM, *Chelonia mydas*) and (f) hawksbill (EI, *Eretmochelys imbricata*) over the nesting seasons – points represent yearly nest counts; trend lines derived from Negative Binomial regression analysis, describe the nesting trends; and shaded areas around these lines indicate 95% confidence intervals.

Green and hawksbill turtles nested year round on São Tomé, with a marked peak in January and February. Nesting activities were notably reduced from April to September for green turtles and April to November for hawksbill turtles (Figure 1a,b). Green turtles were observed nesting on the majority of the island's beaches, with higher nesting in the Caué District in the south, accounting for 90% of the total nests (Figure 1c). A similar preference for the Caué District was shown by hawksbill turtles, with 94% of the nests concentrated there. The Rolas Islet was a favoured nesting site for hawksbill turtles (Figure 1d).

Negative Binomial regression analysis identified an upward trend in nesting for both species. For green turtles, a positive but not statistically significant increase in annual nests was revealed (coefficient = 0.13631, *p* = .0625), suggesting a trend that merits continued monitoring. In contrast, hawksbill turtle nests showed a statistically significant annual rise (coefficient = 0.07855, *p* = .0341). Excluding 2015 data for hawksbills yielded a stronger model fit (AIC = 77.935), affirming the ongoing growth of this nesting population.

### 3.2 Breeding female morphometrics

**FIGURE 2:**
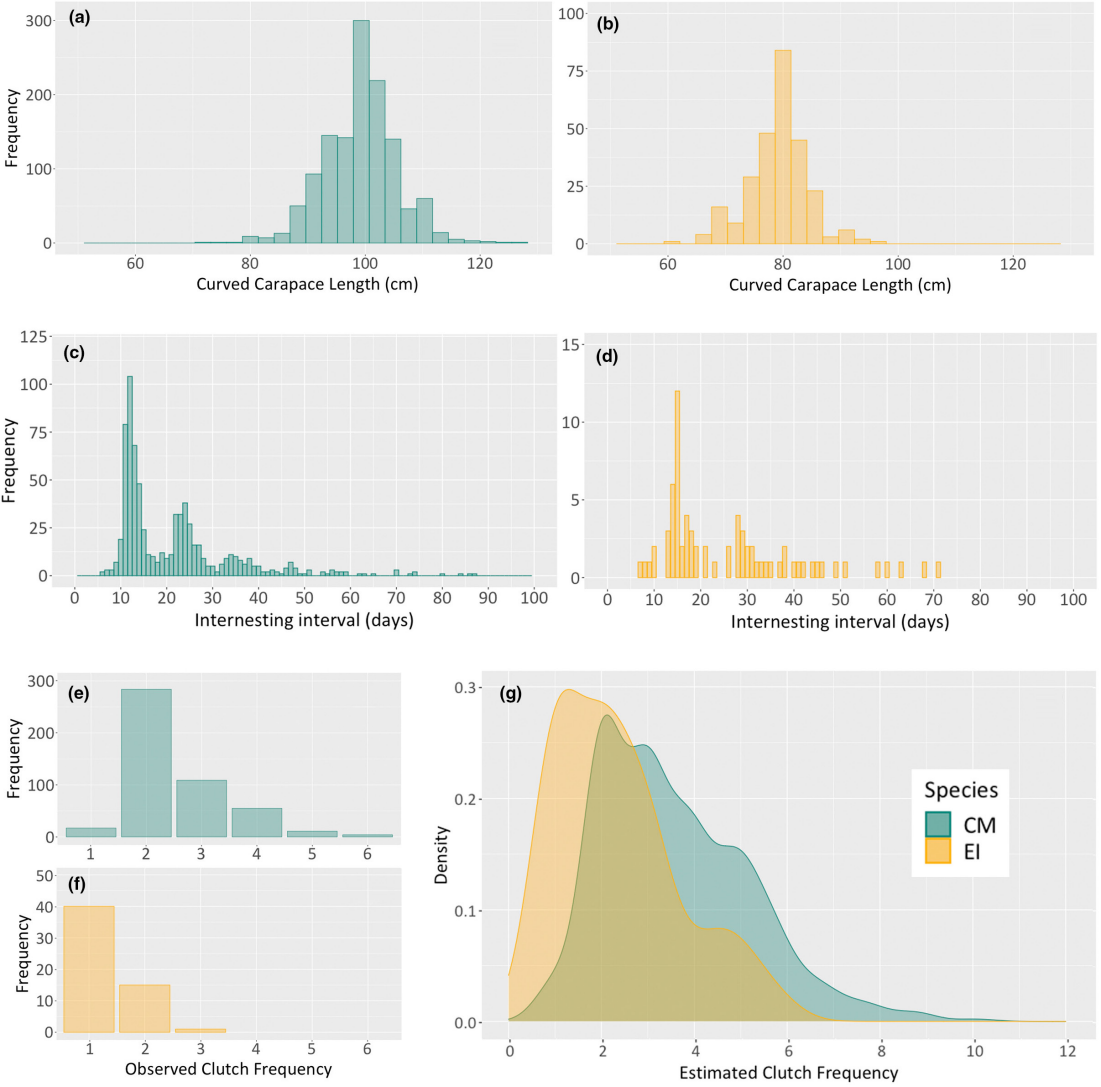
Size‐class frequency distribution of curved carapace length (CCL), inter‐nesting period modal distribution and observed clutch frequency for female green (a, c, e), respectively and for female hawksbill turtles (b, d, f) respectively. The density plot of estimated clutch frequency for the females nesting at São Tomé between 2014 and 2022 (g) is represented as green for green turtles (CM) and yellow for hawksbills (EI).

Descriptive reproductive data for nesting green and hawksbill turtles in São Tomé from 2014 to 2022 are presented in Tables 1 and 2. The mean CCL (cm) of nesting green turtles at first capture was 99.1 cm (SD = 6.36; *n* = 1248; range = 72.3 to 127.0 cm; Figure 2a), whereas for hawksbill turtles it was 79.1 cm (SD = 5.2; *n* = 272; range = 60 to 97 cm; Figure 2b). Over the study period (2014 to 2022), nesting green turtles became larger (Kruskal‐Wallis test: χ^2^ = 103.8, df = 8, *p* < .001), with average carapace sizes in 2022 of 100.1 cm, versus 96.6 cm in 2014. This was not the case for hawksbill turtles though (Kruskal–Wallis test: χ^2^ = 3.09, df = 8, *p* = .9283).

**TABLE 1:** Descriptive reproductive data of green turtles nesting in São Tomé from 2014 to 2022.

Parameter	*N*	Mean ± SD	Range
Females (*n* year^−1^)	6092 (clutches)	212 ± 112	70–348
Nests (*n* year^−1^)	6092 (clutches)	762 ± 404	251–1253
CCL (cm)	1248 (measurements)	99.1 ± 6.4	72.3–127
Inter‐nesting period (days)	265 (individuals)	12.5 ± 1.9	6–18
OCF (nests female)	480 (individuals)	2.5 ± 0.9	1–6
ECF (nests female)	480 (individuals)	3.6 ± 1.6	1–10
Remigration interval (years)	31 (individuals)	2.3 ± 1.5	1–6
Clutch size (eggs)	3330 (clutches)	117.3 ± 22.7	31–261
Nest depth (cm)	381 (clutches)	52.1 ± 11.1	13–93
Emergence success (%)	2547 (clutches)	86.8 ± 12.3	0–100
Incubation period (days)	1964 (clutches)	65.9 ± 8.9	41–103

*Note*: Estimated number of females per year based on estimated clutch frequency.

Abbreviations: CCL, Curved carapace length; ECF, Estimated clutch frequency; OCF, Observed clutch frequency.

**TABLE 2:** Descriptive reproductive data of hawksbill turtles nesting in São Tomé from 2014 to 2022.

Parameter	*N*	Mean ± SD	Range
Females (*n* year^−1^)	1548 (clutches)	84 ± 35	17–123
Nests (*n* year^−1^)	1548 (clutches)	194 ± 81	39–282
CCL (cm)	272 (measurements)	79.1 ± 5.2	60–97
Inter‐nesting period (days)	30 (individuals)	14.6 ± 2.8	7–19
OCF (nests female)	56 (individuals)	1.3 ± 0.5	1–3
ECF (nests female)	56 (individuals)	2.3 ± 1.3	1–6
Remigration interval (years)	9 (individuals)	3.1 ± 2.4	1–7
Clutch size (eggs)	926 (clutches)	126.2 ± 28.2	25–226
Nest depth (cm)	320 (clutches)	44.1 ± 6.2	10–64
Emergence success (%)	174 (clutches)	86.7 ± 11.5	13–100
Incubation period (days)	138 (clutches)	65.6 ± 8.8	44–94

*Note*: Estimated number of females per year based on estimated clutch frequency.

Abbreviations: CCL, Curved carapace length; ECF, Estimated clutch frequency; OCF, Observed clutch frequency.

### 3.3 Inter‐nesting interval and clutch frequency

In our observational estimates for green turtles, inter‐nesting intervals in the unfiltered data ranged from 6 to 87 days (*n* = 723 intervals from 458 individuals). The apparent multimodal pattern (Figure 2c) suggests that the inter‐nesting interval likely ranges from 6 to 18 days, with a mean interval of 12.5 ± 1.9 days (*n* = 265; intervals greater than 18 days are presumably indicative of unobserved nesting events). While for hawksbills turtles, unfiltered inter‐nesting intervals ranged from 7 to 71 days (*n* = 74 intervals from 57 individuals). Similarly, as in Figure 2d, likely inter‐nesting intervals were 7–19 days, with a mean interval of 14.6 ± 2.8 days (*n* = 30).

The observed clutch frequency (OCF) from field surveys ranged from 1 to 6 clutches per season (mean = 2.5 ± 0.9, *n* = 480; Figure 2e). The estimated clutch frequency (ECF), calculated to avoid underestimating clutch frequency, ranged from 1 to 10 clutches per season (mean = 3.6 ± 1.6, *n* = 480; Figure 2g). Satellite tracking data revealed that tracked green turtles displayed a mean interval between nesting events of 11.9 ± 0.4 days and a mean clutch frequency of 5.5 ± 1 nests.

For hawksbill turtles, the OCF ranged from 1 to 3 clutches per season (mean = 1.3 ± 0.5, *n* = 56; Figure 2f), and the ECF ranged from 1 to 6 clutches per season (mean = 2.3 ± 1.3, *n* = 56; Figure 2g). Satellite tracking data revealed that hawksbill turtles had a mean interval between nesting events of 14.6 ± 0.8 days and a mean clutch frequency of 3.7 ± 0.8 nests.

### 3.4 Remigration interval

For green turtles in São Tomé, the mean remigration interval could be determined for 31 turtles, and was 2.3 ± 1.5 years (range 1 to 6 years). This represented 2.4% of the total tagged female population. The most common remigration interval was 1 year (observed in 13 individuals), followed by 2 years (5 individuals), 3 years (5 individuals), 4 years (6 individuals) and 6 years (2 individuals). Similarly, for hawksbill turtles, the mean remigration interval was determined for 9 turtles, and was 3.1 ± 2.4 years (range 1 to 7 years), accounting for 3% of the total tagged female population. The most frequent remigration interval observed was 1 year (3 individuals), followed by 2 years (1 individual), and equal observations for both four and 5 years (2 individuals each), with the seven‐year interval being the least observed (1 individual). The variability in remigration intervals likely indicates a combination of individual differences and an artifact from a smaller sample size on the longer remigration intervals. We acknowledge that the limited sample size for hawksbills may affect the precision of these estimates, but it represents the best available data under the current constraints.

### 3.5 Population size estimation

Using estimated clutch frequency and remigration interval from field observations and also from satellite tracking data, we estimated that 6092 clutches were laid by green turtles and 1548 clutches by hawksbills in São Tomé between 2015 and 2022 (excluding 2014 owing to less monitoring effort than in other years). Using field observation data for green turtles, we divided 6092 clutches by the ECF of 3.6 and then again by the remigration interval of 2.3. This provided an estimate of approximately 736 nesting green turtles. For hawksbill turtles, field observation data of estimated clutch frequency (2.3) and remigration interval (3.1) yielded an estimate of 217 nesting females.

The second method is considered more conservative because it relies on direct evidence of nesting using satellite tracking data. Here, we divided 6092 green turtle clutches by the observed clutch frequency of satellite‐tracked females (5.5 clutches), and the remigration interval of 2.3 years. This yielded an estimated total of 482 nesting green turtles. For hawksbill turtles, the observed clutch frequency of the satellite‐tracked females (3.7 clutches) and the remigration interval of 3.1 years resulted in an estimate of 135 nesting females. Therefore, our study suggests that the total number of female green turtles in this population is likely between 482 and 736, whereas, for the hawksbill turtle population, it is between 135 and 217 females.

### 3.6 Inter‐nesting movements

**FIGURE 3:**
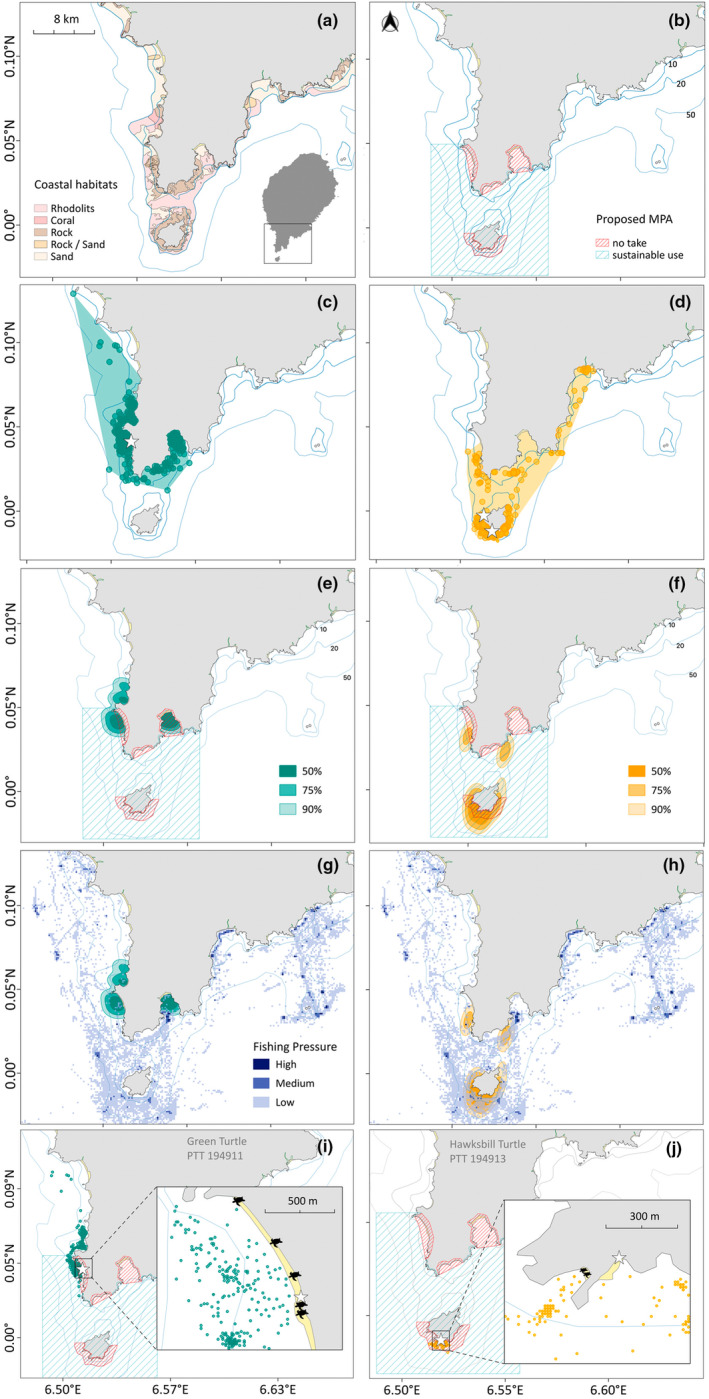
The study site location (a) and marine coastal habitats of the study area (*source*: Airaud et al., 2020); (b) Proposed MPA (PMPA) location of the south of São Tomé, highlighting the no take zone and the sustainable use area (*source*: 4th co‐management assembly of the project establishment of a network of MPAs in São Tomé and Príncipe in a shared management approach managed by the consortium Oikos, FFI, Fundação Príncipe and Marapa, April 20, 2022); Fastloc‐GPS tags locations the minimum convex polygon of the 5 green turtles (c) and the 10 hawksbill turtles (d), the stars indicate the tags deployment locations; autocorrelated kernel density estimations (AKDEs, 50, 75, and 90%) of green (e) and hawksbill (f) turtles tracked during the period of highest nest density for both species during the nesting season of 2019 and 2020 overlapped by the PMPA; AKDEs overlapped by fishing pressure (data collected between 2016 and 2018 on the locations of fishing vessels during fishing activity of the fishing gears most likely to impact green (g) and hawksbill (h) sea turtles: purse‐seine, longline, troll line, surface and bottom gillnet; (i, j) represent one single green and hawksbill turtle, respectively, with their inter‐nesting locations overlapping the PMPA and the deployment location (white star) and precedent nesting locations (black turtles).

We obtained inter‐nesting tracking data from 15 of the 25 satellite‐tagged turtles equipped with Fastloc‐GPS tags, including five green turtles and ten hawksbill turtles. The remaining ten turtles were excluded due to insufficient locations after filtering (*n* = 7) or because they migrated immediately after tag deployment (*n* = 3). The study area, including the coastal habitats and the proposed boundaries of a Marine Protected Area (MPA) south of São Tomé (Jalé‐Rolas‐Malanza), is illustrated in Figure 3a,b, respectively, providing a visual representation of the habitats and areas analysed in this study (see Airaud et al., 2020 for detailed coastal habitat descriptions).

During the inter‐nesting period, the area occupied was 42.3 km^2^ for green turtles and 43.4 km^2^ for hawksbill turtles (Minimum Convex Polygon estimates, MCPs; Figure 3c,d). Throughout the inter‐nesting period, both green and hawksbill turtles primarily remained within 1‐2 km of the coast, mostly over the narrow continental shelf (less than 20 m deep, accounting for 99% of locations). Tagged green turtles nesting on Jalé Beach had core areas (50% home range) covering 1.8 km^2^, which overlapped by 93% with the proposed Marine Protected Area (Figure 3e). For hawksbill turtles nesting at Rolas islet, their core areas covered 1.4 km^2^, which overlapped 100% with the proposed MPA (Figure 3f). However, these core areas were also exposed to artisanal fisheries, with 40% of the green turtle locations and 93% of hawksbill turtle locations potentially impacted (Figure 3g,h).

For green turtles, the minimum residence time in the inter‐nesting area varied from 36 to 62 days, with a mean of 54.5 ± 11.8 days (Table 3, *n* = 4; one turtle was excluded due to premature device failure). Notably, green turtles exhibited a strong tendency to return to the same nesting beach, with subsequent nesting events typically occurring within a mean distance of 203 m of one another (mean ± 178 m, min‐max: 34–540 m, *n* = 4). An example of this nesting behaviour in one individual is illustrated in Figure 3i. However, among the tracked individuals, one turtle demonstrated both inter‐ and intra‐beach nesting behaviour. This turtle's second nesting event was observed on a different beach, approximately 5 km from the initial site, but returned to the same beach for its third event. The fourth nesting event was again on a different beach, this time 1 km away from the third location, with the fifth and sixth events occurring on the same beach as the fourth. Despite these beach switches, the nests were spatially constrained, often within tens of metres of each other. The mean inter‐nesting distance for this turtle was 1.2 ± 2.2 km, with a range of 0.4 to 5 km. Importantly, regardless of the chosen nesting beach, this individual consistently utilised the same inter‐nesting area, located on average 4.4 km from the nesting site.

**TABLE 3:** Summary tracking data.

Turtle id	Species	DT	CCL (cm)	DD	MD	RL (days)	IP (days)	CF
194908	CM	SPLASH	98	13/11/19	19/12/19	36	11.7	4
194909	CM	SPLASH	99	14/11/19	21/12/19	37	12.3	4
194910	CM	SPLASH	100	14/11/19	11/01/20	58	11.2	6
194911	CM	SPLASH	104	16/11/19	16/01/20	61	12.2	6
194912	CM	SPLASH	98	16/11/19	17/01/20	62	12.0	6
194923	CM	SPOT	88	04/02/20	29/02/20	25	–	–
194927	CM	SPOT	104	15/01/20	01/03/20	46	–	–
194928	CM	SPOT	87	16/01/20	29/01/20	13	–	–
194906	EI	SPLASH	80.2	12/11/19	31/12/19	49	16.3	4
194907	EI	SPLASH	81	13/11/19	09/01/20	57	14.0	5
194913	EI	SPLASH	80	09/12/19	07/01/20	29	14.5	3
194914	EI	SPLASH	75	09/12/19	06/01/20	28	14.0	3
194915	EI	SPLASH	78.5	09/12/19	06/01/20	28	14.0	3
194916	EI	SPLASH	81	10/12/19	21/01/20	42	13.7	4
194917	EI	SPLASH	76	11/12/19	09/02/20	60	15.0	5
194918	EI	SPLASH	79.5	12/12/19	27/01/20	46	15.0	4
194919	EI	SPLASH	74	12/12/19	13/01/20	32	15.5	3
194920	EI	SPLASH	83	09/01/20	06/02/20	28	14.0	3
194921	EI	SPOT	75	10/01/20	10/02/20	31	–	–
194922	EI	SPOT	80	10/01/20	11/01/20	1	–	–
194924	EI	SPOT	80	06/02/20	06/02/20	0	–	–
194925	EI	SPOT	82	26/01/21	12/02/21	17	–	–
194926	EI	SPOT	78	26/01/21	29/01/21	3	–	–
194929	EI	SPOT	75	27/01/21	19/02/21	24	–	–
194930	EI	SPOT	90	03/02/21	17/02/21	15	–	–

Abbreviations: CM, *Chelonia mydas*; EI, *Eretmochelys imbricata*; DT, Device Type ‐ SPLASH10‐F‐385A, SPOT‐375B; CCL, Curved Carapace Length; DD, Deployment Date (day/month/year); MD, Migration Date (day/month/year); RL, Residence Length; IP: Inter‐nesting Period; CF, Clutch Frequency per female.

Hawksbill turtles stayed in the inter‐nesting area for 28 to 60 days (mean = 39.9 ± 12.6 days, *n* = 10, Table 3). Hawksbills displayed varied behaviours, with some turtles moving between the nesting beaches and the same inter‐nesting area (6.9 ± 8.9 km away, min–max: 0.5–20 km, *n* = 4), while others used different inter‐nesting areas between each nesting event (4.4 ± 5.0 km away, range 1.2–14.2 km, *n* = 6). Hawksbills revealed a range of nesting behaviours, with most re‐nesting on the same beach (mean distance of 87 m from the previous nest ±116 m, range 15–350 m, *n* = 6). An example of this nesting behaviour in one individual is illustrated in Figure 3j. However, others nested on different beaches after deployment (between 0.2 and 14.2 km away, mean = 4.5 km ± 6.5 SD, n = 4), with subsequent nesting events taking place on the same beach. See Figure S1 for additional insights into the movements of individual turtles.

### 3.7 Diving behaviour

**FIGURE 4:**
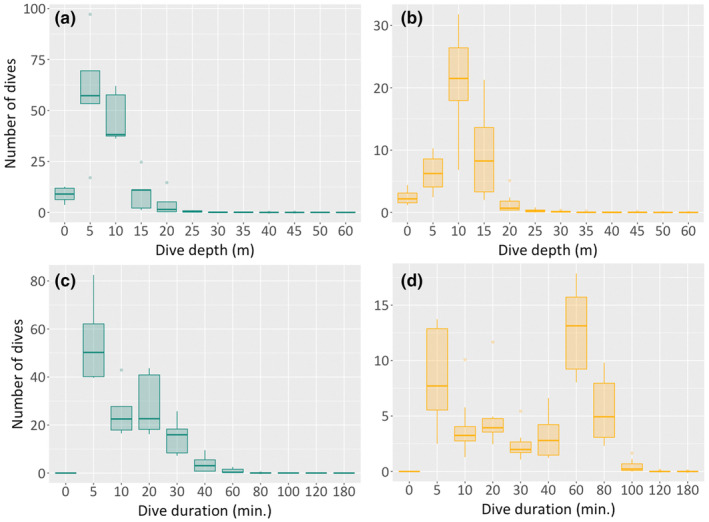
Diving variables recorded by the Fastloc GPS tags show dive depth and duration for green (a, c) and hawksbill (b, d) turtles.

Green and hawksbill turtles exhibited distinct dive behaviours during the inter‐nesting period. Green turtles remained primarily in shallower waters, with the majority (81.5%, *n* = 526) of dives occurring between 5 and 10 m depths (range 5 to 50 m, median 7.9 m, *n* = 645; Figure 4a). Dives deeper than 15 m were less frequent, accounting for 12% (*n* = 76) of total dives. Hawksbill turtles carried out most of their dives (89.4%, *n* = 365) within the 5 to 15 m depth range (mean 10.3 m, range 5 to 50 m, *n* = 411; Figure 4b). Like green turtles, dives exceeding 15 m depth were relatively infrequent (4.6%, *n* = 19).

The majority of dives by green turtles lasted between 5 and 20 min (84.4%; *n* = 544), with a mean duration of 13.7 min and a range of 5 to 180 min (*n* = 645; Figure 4c). Dives exceeding 40 min were rare, constituting only 3.8% (*n* = 25) of total dives. However, for hawksbill turtles, their dive duration distribution varied significantly, with 20.6% (*n* = 84) lasting 5 min and 44.4% (*n* = 182) between 60 and 80 min (mean 39.5 min, range 5 to 180 min, Figure 4d).

**FIGURE 5:**
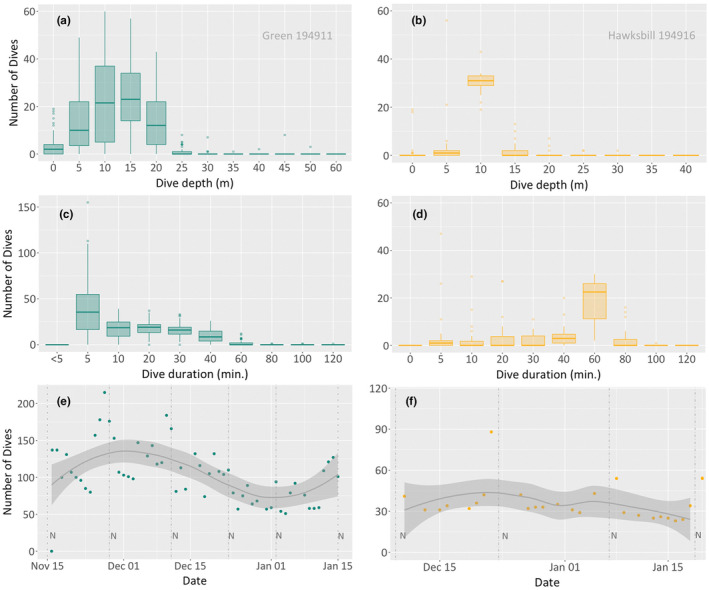
Diving variables recorded by the Fastloc GPS tags showing dive depth and dive duration for one individual green (a, c) and hawksbill (b, d) turtles. (e, f) represent the number of dives during the inter‐nesting period, highlighting each nesting event for each individual (*N*), including a loess smoothing line for trend visualisation, complemented by a grey‐shaded confidence interval to indicate the variability of the data. The green turtle (PTT194911) performed six nesting events, and the hawksbill (PTT194916) performed four nesting events.

**FIGURE 6:**
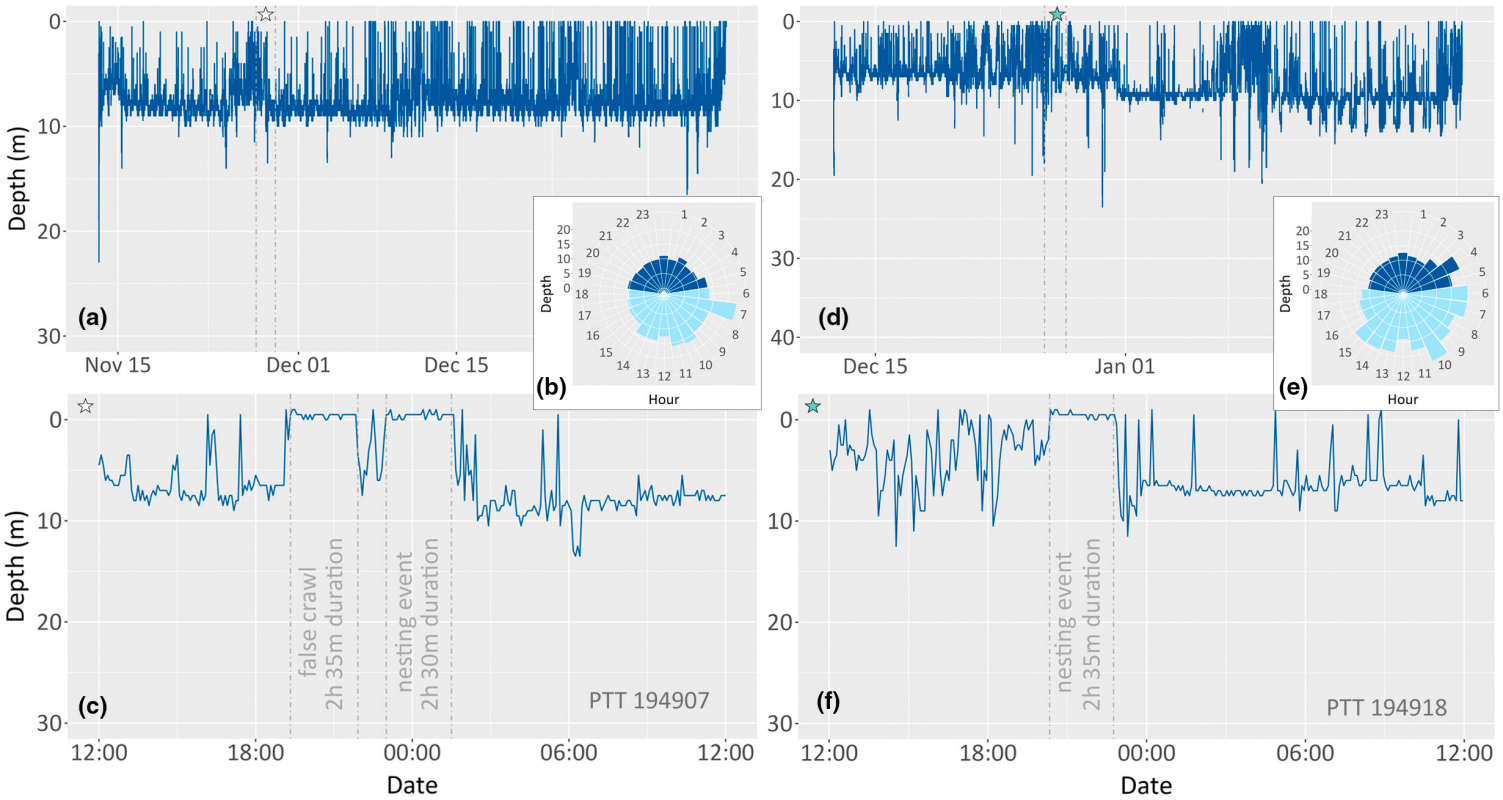
Daily diving frequency of two hawksbills (PTT194907 and PTT194918) with complete data archives (a, d). (c, f) represent the diving behaviour during a nesting event. (b, e) the mean dive depth during the 24‐h range, light blue represents the day (6 to 18 h) and dark blue represents the night (18 to 6 h).

Green turtles performed between 100 and 200 dives daily, whereas hawksbill turtles completed between 20 and 40 dives daily. For both species, there was a general increase in dive frequency across the inter‐nesting interval, with the fewest dives typically taking place immediately after nesting and a substantial increase on the day before the next nesting event. An example of this diving behaviour in individual green and hawksbill sea turtles is illustrated in Figure 5. Individual nesting events could be observed in the data from two hawksbill turtles from which satellite tags were retrieved, with complete data archives (Figure 6).

## 4 DISCUSSION

This study is the first to survey the nesting behaviour, including near‐shore telemetry for hawksbill and green turtles in the Gulf of Guinea. It provides a novel perspective on the behaviours of these less‐studied turtle populations in the Gulf of Guinea: a genetically important green turtle population (Hancock, 2019) and, importantly, the largest hawksbill rookery in the Eastern Atlantic (Ferreira‐Airaud et al., 2022).

Both species predominantly nest from September through April in São Tomé, similar to other rookeries in the southern hemisphere (Almeida et al., 2011; Bellini et al., 2013; Marcovaldi et al., 1999, 2007; Weber et al., 2014). This period coincides with São Tomé's rainy season (Ceríaco et al., 2022), which recent studies suggest is experiencing a trend of increasing precipitation, particularly in the southern parts of the island (Chou et al., 2020). The rainy season can alter beach conditions, such as sand temperature and humidity, which are crucial for nest construction and may influence demographic parameters like the sex ratio (Laloë et al., 2021; Lolavar & Wyneken, 2015). Despite these potential challenges, we observed high nest emergence success rates for both green (86.8 ± 12.3%) and hawksbill (86.7 ± 11.5%) turtles, suggesting that these species are well adapted to the local rainy season conditions. However, with the potential for increased rainfall due to climate change, the impact of these changes on nesting success remains an area for future research. The varied environmental conditions across São Tomé, including differential rainfall and beach characteristics, might be expected to influence the spatial distribution of nesting sites. For example, turtles might show preferences for certain locations that offer optimal conditions for nest success, a consideration that could become increasingly important as climate change affects rainfall patterns.

Both green and hawksbill turtles nesting on São Tomé are among the smallest reported in the Atlantic, with respective mean curved carapace lengths of 99.1 ± 6.4 cm and 79.1 ± 5.2 cm. These small sizes at maturity could reflect the impact of years of harvest (Balazs & Chaloupka, 2004; Piacenza et al., 2016; Van Houtan & Kittinger, 2014). However, other factors such as physical and biological ecosystem characteristics, food availability and migration patterns might also influence mean turtle size (Chaloupka et al., 2004; del Monte‐Luna et al., 2012; Snover et al., 2010).

The relatively high proportion of neophytes (young adults) in the reproductive population, as seen in expanding populations, suggests that not enough time has passed to observe an increment in size (López‐Castro et al., 2022). As these neophytes mature, the average size of nesting females is expected to increase, aligning with the recovery and stabilisation of the population (Hays et al., 2022). Ecological changes in foraging habitats, potentially driven by climate change, may also play a role. Studies have indicated a reduction in body size in sea turtle populations due to changes in ocean productivity and foraging habitat quality, which can affect reproductive output and overall fitness (Bjorndal et al., 2016, 2017; Le Gouvello et al., 2020; Stephenson, 2014). In our study, the observed increase in the size of green turtles may reflect the recruitment of younger individuals. However, given the limited duration of our study, it is crucial to acknowledge that this timeframe may not fully capture long‐term trends in turtle sizes and their broader ecological implications. To gain a deeper understanding, it is essential to revisit this study in the future, allowing for a more extended observation period. Meanwhile, a critical step involves thoroughly investigating the current state of foraging areas for hawksbill and green turtles nesting in São Tomé. Undertaking comprehensive spatial analyses to identify key environmental drivers and develop effective strategies for habitat conservation will be essential.

In the present study, we were able to make the first estimate of the population size of both species nesting in São Tomé. Using satellite‐tracking method to provide estimates of nesting activity, along with information on remigration interval, we estimated a population of 482 green turtles and 135 hawksbills, whereas the field observation‐based method gave us higher population estimates, with 736 green turtles and 217 hawksbills. We recognise that both methods come with inherent uncertainties and potential biases. For example, satellite tracking data may not always capture all nesting events of an individual during the nesting season. Observational errors and environmental variability could impact the estimated clutch frequency obtained from field observations. Despite this, our approach is still likely to be useful in coarsely estimating the population size. In our analysis of sea turtle nesting in São Tomé Island, it is important also to consider the contribution of nesting sites on Príncipe. Recent studies, such as Ferreira‐Airaud et al., 2022, indicate that Príncipe likely hosts a significant portion of the nesting population: approximately 30% of hawksbill and 65% of green turtles nesting in the entire archipelago. Taking into account this distribution, we estimate the overall population size to be between 170 and 300 female hawksbills and 800 and 1200 female green turtles nesting across both São Tomé and Príncipe. This estimate is critical for designing effective conservation strategies that ensure these species' continued survival and recovery in São Tomé. In particular, both green and hawksbill turtles remain close to the shore throughout the inter‐nesting period, overlapping with artisanal fisheries. This underscores the urgent need for enforcement of Marine Protected Areas (MPAs), with a combination of no‐take zones and seasonal spatial/temporal closures, considering local socio‐economic needs and considerations (Scott et al., 2012).

A notable contrast was observed in the diving behaviours of green and hawksbill turtles during the inter‐nesting period. Green turtles predominantly made short, shallower dives within the 5–10 meter range, while hawksbill turtles exhibited a mix of short and longer shallow dives (as described elsewhere in the Atlantic and Pacific Oceans, Bell & Parmenter, 2008; Gaos et al., 2012; Blanco et al., 2013; Walcott et al., 2013; Chambault et al., 2016; Monteiro, 2022). These behavioural patterns highlight a pronounced use of shallower waters by both green and hawksbill turtles. In terms of management implications, adjusting artisanal fishing practices, particularly the geographical location of net deployment, could mitigate the impact on turtle populations. Shifting fishing activities away from these shallower areas to deeper waters could feasibly reduce turtle bycatch. Importantly, this approach aims to balance turtle conservation with the preservation of local fishing communities' livelihoods. By relocating fishing efforts to less turtle‐dense areas that remain within productive fishing zones, the overall catch efficiency for fishermen might not be significantly affected. It's essential, however, to engage in a collaborative process with these communities to assess and refine this strategy, ensuring it aligns with both conservation goals and socio‐economic sustainability. Conservation measures must, therefore, be informed by a nuanced understanding of species‐specific behaviours and habitat preferences, alongside a thorough consideration of the local socio‐economic context, to enhance their effectiveness while minimising unintended impacts on both vulnerable sea turtle populations and the livelihoods of local communities.

The observed dive patterns in our study suggest potential transitions between the inter‐nesting area and the nesting beach, as well as varying energy‐saving strategies (Fossette et al., 2008; Hochscheid et al., 1999; Thomson et al., 2011). Green turtles, primarily herbivorous (Ballorain et al., 2010), were found in areas lacking their usual food sources like seagrass (Airaud et al., 2020), which might imply either a reliance on alternative food sources or fasting during this period. Conversely, hawksbill turtles, known for a varied diet including marine invertebrates and algae (Bell, 2013; León & Bjorndal, 2002), exhibited diverse diving behaviours. Although these patterns could be indicative of foraging, it is also well‐documented that fasting is a common strategy among sea turtle populations during the inter‐nesting period, a time characterised by high energy expenditure due to reproductive activities (Goldberg et al., 2013; Hays et al., 2002). Interestingly, studies in some regions suggest that green turtles may forage during the nesting season if food is available (Ferreira et al., 2006; Stokes et al., 2019), but similar foraging behaviour during this period has not been documented for hawksbills. The small and shallow platform of São Tomé (Cowburn, 2018; Maia et al., 2018) may also influence the dive depths of both species, possibly providing habitats that serve as resting and/or foraging sites, thereby reducing their energy expenditure (Monteiro, 2022; Walcott et al., 2012). Therefore, while our study presents dive patterns that may indicate resting or potential foraging, the absence of direct feeding observations excludes definitive conclusions about their feeding behaviour during this period. Future research integrating diving pattern analysis with direct observation or additional methodologies is essential to elucidate these behaviours and their ecological implications more clearly.

Our research highlights that turtles occupy a particularly restricted area during the inter‐nesting period. The combined core areas of satellite‐tagged green turtles nesting on Jalé beach covered just 1.8 km^2^, overlapping by 93% with the proposed Marine Protected Area. Similarly, hawksbill turtles nesting at Rolas Islet occupied just 1.4 km^2^, and were completely encompassed by the proposed MPA. These findings indicate that the proposed MPA (pMPA) is probably well‐located to protect green and hawksbill turtles during the inter‐nesting period. However, achieving the conservation potential of the pMPA calls for robust management strategies. In particular, intense fishing activity occurs in the pMPA, so well‐balanced management must balance the dual goals of marine conservation and maintaining local fishing livelihoods, leading to a sustainable coexistence. A crucial observation from our study is that much of the fishing in the pMPA is artisanal, characterised by low‐cost, labour‐intensive fishing from small boats, yet contributing significantly to the overall fishing pressure at a global scale (Metcalfe et al., 2017). In the context of São Tomé and Príncipe, artisanal fisheries are not only a traditional activity but also contribute substantially to local economies, providing 85% of animal protein consumed locally and around 16% of employment of the active population (Sy & Diogo, 2019; FAO, 2021; Zacarias et al., 2022; FAO et al., 2023). Thus, the dual goals of marine conservation and maintaining local fishing livelihoods must be balanced to lead to sustainable coexistence. The interaction between artisanal fishing activities and sea turtle inter‐nesting habitats is particularly complex and warrants further examination. Understanding whether certain fishing practices disproportionately affect critical turtle nesting sites could inform more effective conservation measures. However, recognising the limitations of our current data, we advocate for enhanced monitoring and data collection efforts that could support such detailed analyses in the future, contributing to a symbiotic relationship between artisanal fisheries and marine conservation initiatives (Di Franco et al., 2020; Mascia et al., 2010).

Aside from artisanal fisheries, other anthropogenic factors impact the conservation status of hawksbill and green turtles of São Tomé. The proliferation of illegal sand mining activities (Ferreira‐Airaud et al., 2022; Muñoz‐Torrent et al., 2022) contributes substantially to the erosion of nesting beaches (Anim et al., 2013; Masalu, 2002; Tanner, 2013). When this phenomenon is coupled with climate change projections, such as sea‐level rise (Fuentes et al., 2010; Lyons et al., 2020; Varela et al., 2019) and increased frequency of storm events (Laloë et al., 2021; Rivas et al., 2018), the consequence is a worrying trend of diminishing suitable nesting habitats (Fuentes et al., 2011). Simultaneously, although economically advantageous, the growing tourism industry on São Tomé brings additional conservation challenges. Increased construction of small hotels, often without adequate regulation or environmental impact assessments, places pressure on the nesting beaches (Ferreira‐Airaud et al., 2022), with artificial lights (Brei et al., 2016; Silva et al., 2017), and other threats such as increased human traffic, pollution from waste, and habitat destruction for building, representing serious pressures on local biodiversity (Hall, 2010; Jones, 2022; Meletis & Harrison, 2010). This trend, and inadequate local law enforcement, underlines the need for robust environmental governance. Effective conservation will necessitate the integration of biodiversity protection objectives into broader developmental policies and enforcement of regulatory adherence (Alegre, 2009; Beger et al., 2015; Lima et al., 2022). In light of these pressing concerns, the recent creation of ecological reserves by the national government (Law No. 08/2023), in primary nesting areas for green and hawksbill turtles provides a beacon of hope. If effectively managed, these coastal terrestrial protected zones could function as vital refuges for turtles, potentially mitigating some of the detrimental impacts associated with anthropogenic threats (Maestro et al., 2019; Nickols et al., 2019; Watson et al., 2014).

Despite historically facing intense exploitation (Ferreira‐Airaud et al., 2022), both green and hawksbill turtle populations nesting in São Tomé are showing a progressive trend, reflecting recovery patterns observed in other rookeries (Barbosa et al., 2018; Blumenthal et al., 2021; Hudgins et al., 2023; Kauffman, 2022; Mazaris et al., 2017; Restrepo et al., 2023; Santos et al., 2013; Seminoff et al., 2015). The encouraging positive trends observed in both species are indicative of the substantial impact of conservation efforts implemented in the region in recent years. Local conservation initiatives, such as Programa Tatô (established in 1998), marked a concerted effort to reverse the declining trends of sea turtle populations in São Tomé (Formia et al., 2003; Graff, 1996; Rosseel, 1997). However, it was only in 2014 that the program evolved to incorporate an integrative approach, significantly amplifying its impact (Ferreira‐Airaud et al., 2022; Vieira et al., 2016). Since then, Programa Tatô has brought together a wide array of stakeholders, and has been monitoring sea turtle populations, fostering alternative livelihoods to reduce dependency on sea turtle harvesting, encouraging law enforcement strategies, and enhancing awareness about sea turtle conservation. This inclusive and synergistic approach, combined with focused research and monitoring and protection of critical nesting sites, is likely a major driving force behind the positive trends observed in the nesting behaviours of both green and hawksbill turtles on São Tomé.

In conclusion, our study provides invaluable insights into the behaviour, nesting patterns, and population trends of sea turtles in São Tomé. It also highlights the importance of systematic, long‐term monitoring for assessing the status of these species and shaping effective local and regional conservation strategies. The observed recovery in the green and hawksbill turtle populations in São Tomé showcases the positive impact that well‐coordinated, holistic and adaptive conservation programmes like Programa Tatô can achieve, setting a valuable precedent for other regions globally. Guiding principles for future conservation efforts should encompass science‐based management practices and a commitment to balancing anthropogenic needs with biodiversity preservation.

## AUTHOR CONTRIBUTIONS

**Betânia Ferreira‐Airaud:** Conceptualization (lead); data curation (lead); formal analysis (lead); funding acquisition (lead); investigation (lead); methodology (lead); project administration (equal); resources (lead); software (lead); supervision (lead); validation (lead); visualization (lead); writing – original draft (lead). **Sara Vieira:** Conceptualization (equal); data curation (equal); funding acquisition (supporting); investigation (supporting); methodology (equal); project administration (supporting); validation (supporting); visualization (supporting); writing – original draft (supporting). **Maria Branco:** Data curation (equal); validation (supporting); visualization (supporting). **Antunes Pina:** Data curation (supporting); validation (supporting); visualization (supporting). **Venceslau Soares:** Data curation (supporting); validation (supporting); visualization (supporting). **Manjula Tiwari:** Conceptualization (supporting); methodology (supporting); resources (supporting); validation (supporting); visualization (supporting); writing – review and editing (equal). **Matthew Witt:** Formal analysis (supporting); supervision (supporting); validation (supporting); visualization (supporting); writing – review and editing (equal). **Rita Castilho:** Supervision (equal); validation (equal); visualization (equal); writing – review and editing (equal). **Alexandra Teodósio:** Supervision (equal); validation (equal); visualization (equal); writing – review and editing (equal). **Lucy A. Hawkes:** Conceptualization (equal); formal analysis (equal); investigation (equal); methodology (equal); supervision (lead); validation (equal); visualization (equal); writing – original draft (equal).

## CONFLICT OF INTEREST STATEMENT

BF‐A, SV and MB are pro‐bono members of the Board of Directors of Associação Programa Tatô, and VS and AP are employees of Associação Programa Tatô. There are no other conflicts of interest to declare.

## Supporting information


Figure S1.

## Data Availability

The research was undertaken by Programa Tatô, an organisation duly authorised by the Ministry of Environment of São Tomé and Príncipe (Environment and Climate Action Directorate), to perform monitoring, research, and protection activities related to sea turtles and their habitats in São Tomé and Príncipe. The datasets supporting this article are accessible in the following public repositories: nesting data can be found in the Dryad Digital Repository and tracking data in the Movebank database. For additional information or data‐related inquiries, please contact the corresponding authors.
