# Supplementary material for: Green and Hawksbill Sea turtles of Eastern Atlantic: New insights into a globally important rookery in the Gulf of Guinea
Source: Ecol Evol. 2024 Mar 18;14(3):e11133. doi: 10.1002/ece3.11133 (PMC10948591; doi:10.1002/ece3.11133)

Figure S1 Daily Fastloc-GPS tags locations of five green individual turtles and ten hawksbill individual turtles during the nesting season of 2019 and 2020


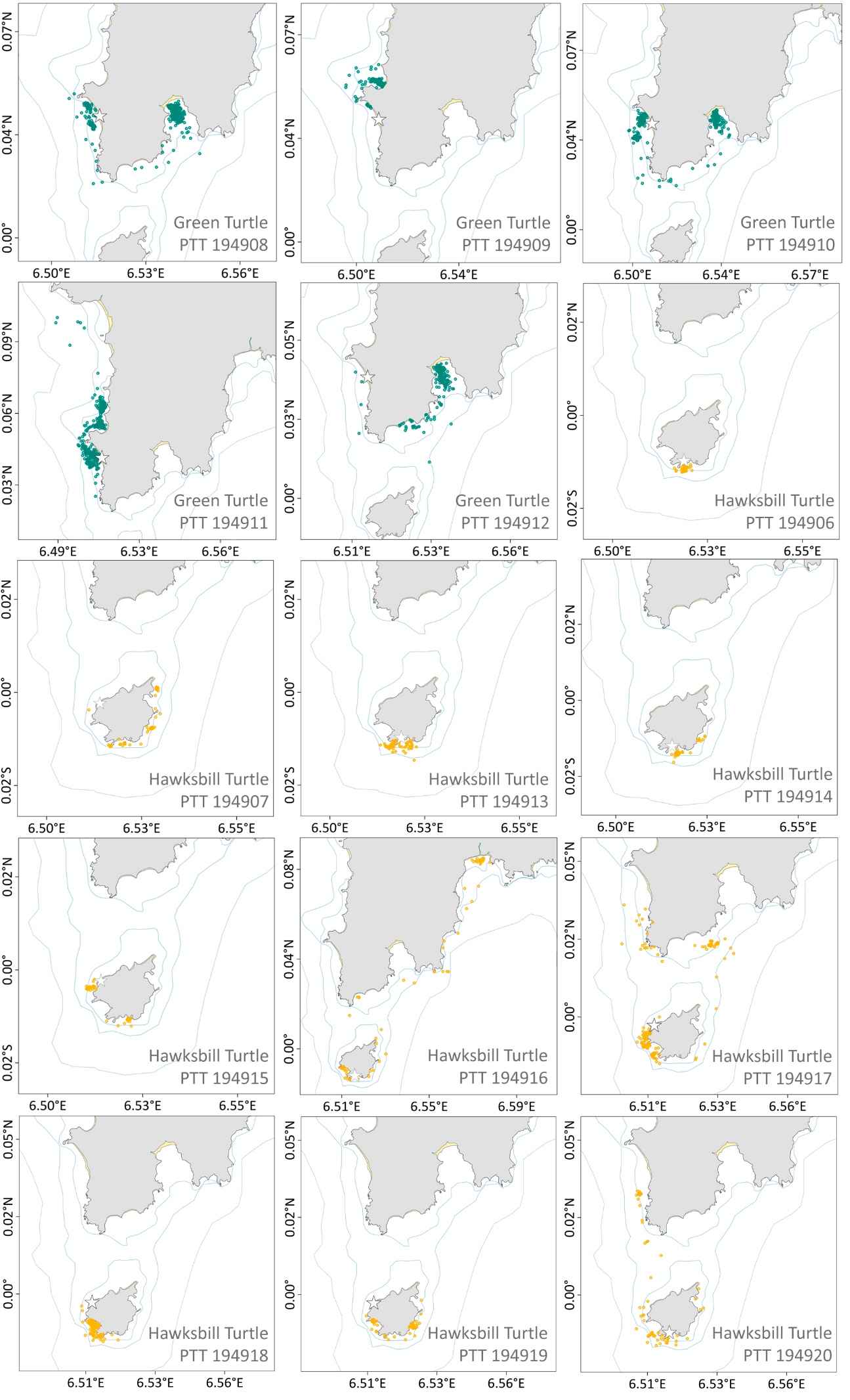

Supplement: Supplementary file 1 — Figure S1. [file ECE3-14-e11133-s001.docx]
